# Advances in preparation and application of antibacterial hydrogels

**DOI:** 10.1186/s12951-023-02025-8

**Published:** 2023-08-26

**Authors:** Yixin Tang, Huiqing Xu, Xue Wang, Shuhan Dong, Lei Guo, Shichen Zhang, Xi Yang, Chang Liu, Xin Jiang, Mujie Kan, Shanli Wu, Jizhou Zhang, Caina Xu

**Affiliations:** 1 Department of Biochemistry, College of Basic Medical Sciences, Jilin University, Changchun, 130021 Jilin China; 2 Department of Preventive Medicine, School of Public Health, Jilin University, Changchun, 130021 Jilin China; 3 Department of Oral and Maxillofacial Surgery, Hospital of Stomatology, Jilin University, Changchun, 130021 Jilin China

**Keywords:** Chemical cross-linking, Antibacterial hydrogels, Light-mediated hydrogels, Antibacterial properties, Biomedical application, Drug delivery systems, Infection treatment

## Abstract

Bacterial infections, especially those caused by drug-resistant bacteria, have seriously threatened human life and health. There is urgent to develop new antibacterial agents to reduce the problem of antibiotics. Biomedical materials with good antimicrobial properties have been widely used in antibacterial applications. Among them, hydrogels have become the focus of research in the field of biomedical materials due to their unique three-dimensional network structure, high hydrophilicity, and good biocompatibility. In this review, the latest research progresses about hydrogels in recent years were summarized, mainly including the preparation methods of hydrogels and their antibacterial applications. According to their different antibacterial mechanisms, several representative antibacterial hydrogels were introduced, such as antibiotics loaded hydrogels, antibiotic-free hydrogels including metal-based hydrogels, antibacterial peptide and antibacterial polymers, stimuli-responsive smart hydrogels, and light-mediated hydrogels. In addition, we also discussed the applications and challenges of antibacterial hydrogels in biomedicine, which are expected to provide new directions and ideas for the application of hydrogels in clinical antibacterial therapy.

## Introduction

At present, human has paid great attention to infections caused by bacteria the infections caused by drug-resistant bacteria has posed a great threat to human being health [1–3]. As is known to all, humans have been plagued by influenza pandemics, which have brought misery, disease, and even death to humans over the past few hundred years [4]. Secondary bacterial infections due to influenza are associated with greatly increased mortality, particularly some infections caused by gram-positive bacteria such as *Streptococcus pneumonia* and *Staphylococcus aureus* (*S. aureus*) [5, 6]. Since the discovery of penicillin by Alexander Fleming, the use of antibiotics provided a new method of antibacterial treatment [7, 8]. However, abuse of antibiotics has resulted in the occurrence of multidrug-resistant bacteria, for example, methicillin-resistant *Staphylococcus aureus *(MRSA), vancomycin-resistant *Enterococcus* (VRE) [9], and some superbugs [10], which are difficult to deal with, have seriously threatened our health. Research studies have shown that if we do not provide treatment strategies for drug-resistant bacteria, this would result in 700,000 deaths from infectious diseases and a loss of 10 billion dollars per year worldwide [11, 12]. Therefore, it is urgent to develop new antibacterial strategies or therapeutic methods for improving the antibacterial effects.

With the development of research, researchers have found that inorganic antibacterial agents, organic antibacterial agents, and hydrogel-based agents exhibit good antibacterial activity, providing the new strategies for the treatment of bacterial diseases [13–15]. Among them, hydrogel, as a kind of good antibacterial materials, can be used with a variety of antibacterial agents to achieve antibacterial treatment [16]. Hydrogels are a kind of hydrophilic polymers with a three-dimensional porous structure, formed by physical or chemical cross-linking of polymer chains [17, 18]. Hydrogels are soft in texture, high water-holding capacity, and have good biocompatibility close to living tissue [19], and have potential applications in biomedicine [20], such as cell culture [21], drug delivery and loading [22, 23], tissue engineering [24, 25] and other medical fields. Hydrogels have significant advantages in antibacterial materials due to their efficient loading, effective release of drugs and antibacterial agents, thus greatly improving the utilization of antibacterial agents and reducing the toxic effects of antibacterial agents on cells [26, 27]. Thus, hydrogels have attracted increasing attention as alternative materials for the treatment of bacterial infections.

**Fig. 1:**
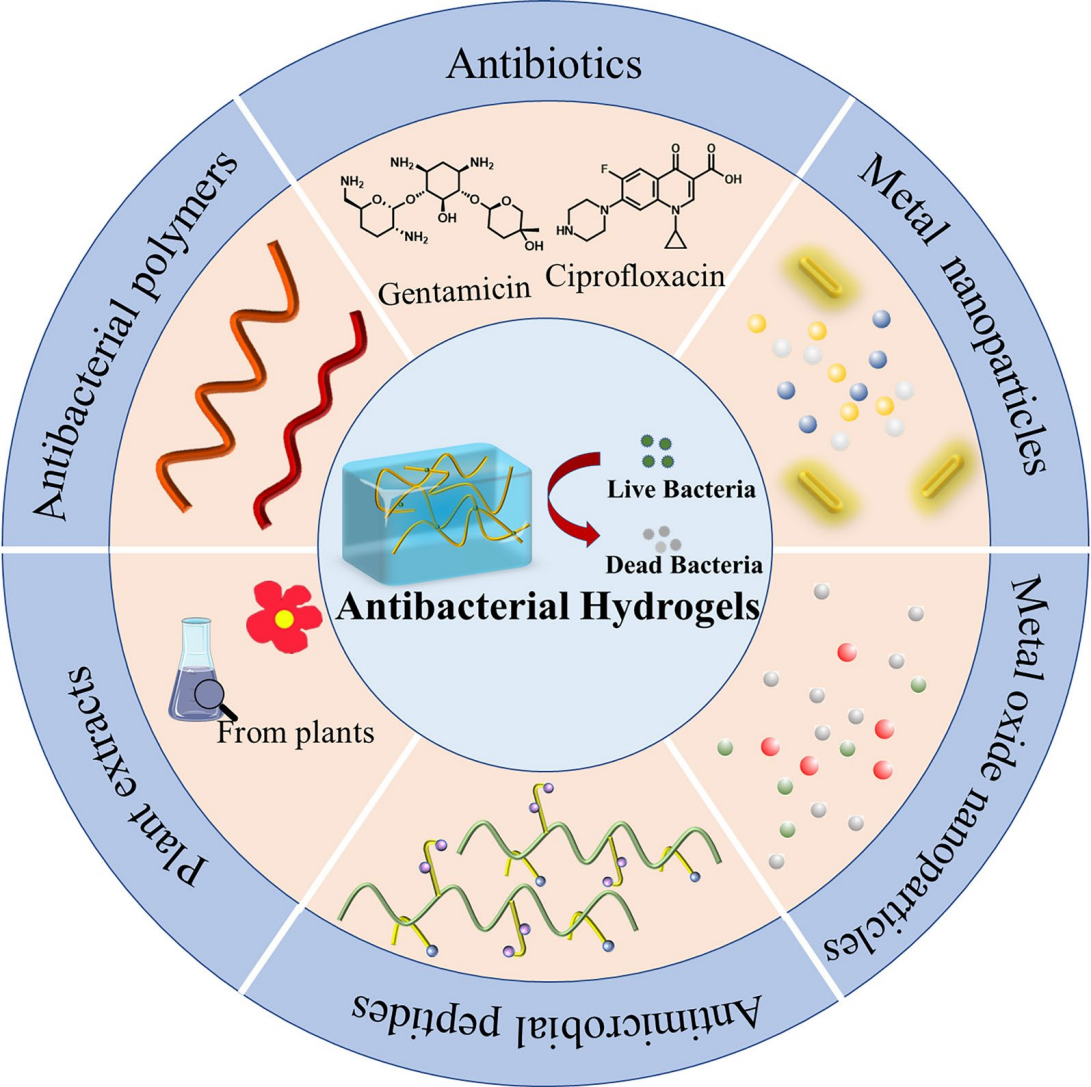
The different antibacterial agents incorporated into hydrogels

However, with the widespread use of conventional hydrogels, bacterial resistance to antibiotics has been increasing, which has caused conventional hydrogels to gradually lose their advantages. Meanwhile many advanced hydrogels have been developed to solve the problem. Therefore, this article summarizes the progress and development of the application of hydrogels in the antibacterial field. According to antibacterial therapy strategies, antibacterial therapy based on hydrogel could be summarized as the following several categories: (I) Antibiotic loaded hydrogels, (II) Antibiotic-free loaded hydrogels, (III) The stimuli-responsive smart antibacterial hydrogels, and (IV) Light-mediated antibacterial hydrogels. Among them, the different antibacterial agents incorporated into the antibacterial hydrogels are shown in Fig. 1. In this review, from the perspective of synthesis processes, properties and mechanisms will be introduced in about antibacterial hydrogel of treatment strategies and we hope to provide direction and basis for reducing the occurrence of bacterial drug resistance and improving the antibacterial strategy of hydrogels.

### Methods for preparing hydrogels

Hydrogels are 3D polymers formed by combining hydrophilic polymers with water molecules [28]. The physicochemical properties of hydrogels are highly dependent on the cross-linking methods. The key to the preparation of hydrogels is the cross-linking method. According to the type of bonds formed by cross-linking between the polymer chains, the preparation techniques of hydrogels can be divided into physical cross-linking method and chemical cross-linking method. The hydrogels formed by physical cross-linking method are called physical hydrogels, which are cross-linked by non-covalent bonding interactions. Physical cross-linking method has the advantages of low cost, low cytotoxicity, and simple operation [29, 30]. Chemical cross-linking method to form hydrogels requires the assistance of polymeric cross-linking agents [31]. Compared with physical cross-linking method, chemical cross-linking has the advantage of forming stronger chemical bonds with a higher degree of cross-linking.

### Physical cross-linking hydrogels

Physical cross-linking is the physical process of forming non-covalent bonds whose products can be reversibly formed or disrupted [32], such as ion interaction [33], hydrophobic interaction [34], and hydrogen bonds. Among them, the polymer alginates are often cross-linked through ionic interaction, which are anionic linear polysaccharides with mannuronic acid residues and can be cross-linked by calcium and barium ions to form physical hydrogels [35, 36]. Alginate-based hydrogels have attracted great attention due to their biocompatibility and low cost [37–39]. Choi et al. mixed negatively charged alginates with positively charged chitosan (CS) to form ion interaction to stabilize the structure. At the same time, β-glucan was added to promote wound healing. It has been reported that alginate-CS hydrogel patches containing β-glucan were expected to be wound dressings with antibacterial properties [40]. Besides, Meng et al. mixed stearyl methacrylate with silk fibroin and cross-linked it with the alginate network to construct silk fibroin-based hydrophobic-association hydrogels [41]. The as-prepared hydrogels by replacing sacrificial bonds with hydrophobic interaction not only enhanced the strength and toughness of the hydrogel, but also achieved self-healing properties. It was worth mentioning that the self-healing process of the composite hydrogel did not require external stimulation at room temperature. More importantly, the strategy of biomolecular preparation of physical hydrogels was adopted, of which collagen was an example [42]. Collagen is a natural polymer that is a major component of the extracellular matrix [43]. Lohrasbi et al. incorporated cellulose nanofibrils into collagen hydrogels to enhance the mechanical properties and maintain the biocompatibility of the hydrogels [44].

### Chemical cross-linking hydrogels

Chemical cross-linking hydrogels are polymers linked by covalent bonds, and some chemical cross-linking agents can be triggered under light and high-energy radiation. Although some chemical agents are toxic to cells, chemical hydrogels form stronger bonds and are more thermally resistant than physical hydrogels [29]. The way to alter the functional and mechanical properties of hydrogels is to add small cross-linking agents such as glutaraldehyde (GA), dopamine, and tannic acid (TA), genipin (GP). Among them, GA, a chemical cross-linking agent, is extensively used to cross-link tissue films and scaffolds for transplantation procedures including heart valve replacement [45]. Due to the toxicity of GA to cells, its application is limited [29]. However, TA is added to polymer networks as an ideal substitute to enhance the function of biomaterials [46]. GP extracted from the fruit of Gardenia is an active ingredient of Chinese herbal medicine for the treatment of liver disease [47]. GP has recently become a popular cross-linking agent because of its good biocompatibility and low cytotoxicity to cells compared with GA [48]. Lu et al. developed an injectable composite collagen hydrogel that was prepared by cross-linking carbon dot nanoparticles (CD NPs) through using GP as a linker [49]. Due to the presence of GP and CD NPs, the stiffness of the hydrogel was enhanced, and photodynamic therapy could generate reactive oxygen species, both of which resulted in improved cartilage differentiation.

In addition to the synthesis of hydrogels using chemical cross-linking agents, Schiff base formation, enzymatic induced cross-linking, photoactivated cross-linking, and radiation cross-linking have also been reported for preparing hydrogels [50]. Among them, photoactivated cross-linking has attracted much attention and has been widely applied in the preparation of cytokine-encapsulated hydrogels [51]. This method can rapidly form hydrogel under mild conditions, while the mechanical properties of hydrogels can be controlled by adjusting the reaction, and the cross-linking sites can be precisely selected [52]. Besides, the covalent reaction of enzymatic cross-linking is mild and can be done in our body without any additional chemical reagents. Therefore, it is non-cytotoxic to cells and more suitable for tissue engineering [53, 54]. It is worth noting that radiation cross-linking reduces the use of cross-linking agents compared to others and can be done under mild conditions [55].

**Fig. 2:**
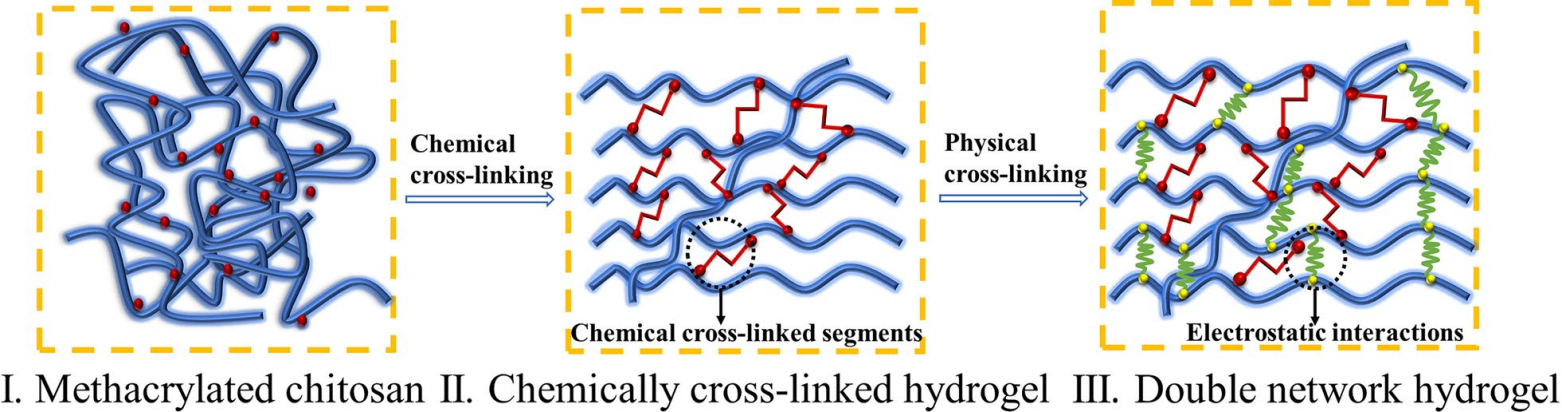
Formation process of double network hydrogel. Reproduced with permission [56]. Copyright 2013 Elsevier Ltd

In addition to physical and chemical cross-linking methods, the third class of hydrogels have been reported to combine both types, termed dual-network hydrogels. As shown in Fig. 2, the process of preparing physical-chemical double network hydrogel was roughly as follows: methacrylated CS formed chemical hydrogels by cross-linking, and then CS interacted with chondroitin sulfate to form physical hydrogels through electrostatic interaction [56]. For example, Liu et al. synthesized thiolate chitosan (CS-NAC) used together with silk fibroin (SF) to form dual network CS-NAC/SF hydrogels [57]. The CS-NAC/SF gels had stronger mechanical properties compared to CS-NAC or SF gels.

## Antibacterial treatments based on hydrogels

### Antibiotic‐loaded antibacterial hydrogels

Over the past few decades, the use of antibiotics as the first choice for antibacterial therapy has been successful in antibacterial therapy [58, 59]. Antibiotics can be divided several categories: fluoroquinolones, beta-lactams, macrolides, tetracyclines, and aminoglycosides. In general, the effective treatments for infections are direct oral or injectable antibiotics. Despite systemic treatment, direct use of antibiotics is difficult to control drug usage, which can lead to adverse factors such as bacterial resistance [60, 61]. The hydrogel has a porous structure suitable for loading antibiotic drugs, allowing direct administration at the site of infection thereby reducing the incidence of antibiotic abuse and improving the utilization of antibiotics while having good biocompatibility [62, 63]. The following was the introduction to the hydrogels loaded with antibiotics.

**Fig. 3:**
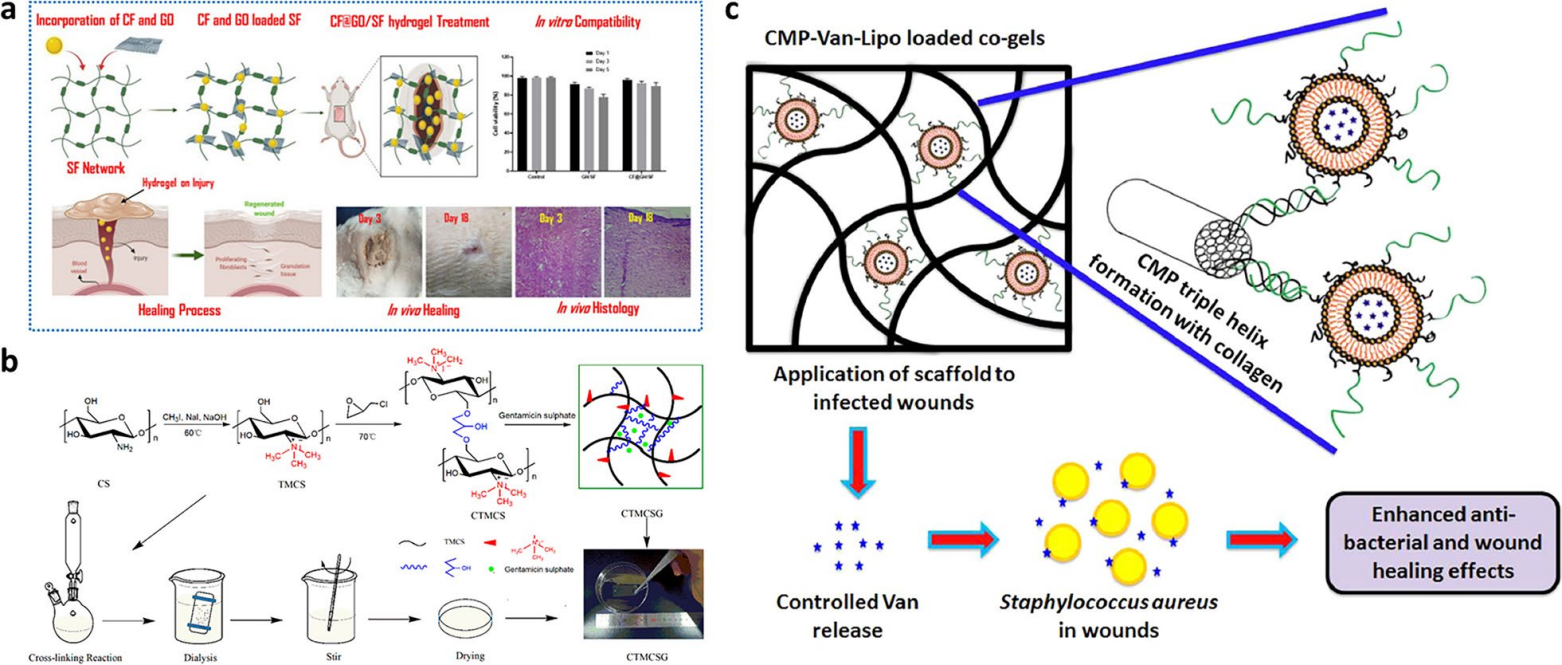
**a** Schematic synthesis of graphene/silk cellulose composite hydrogel loaded with Ciprofloxacin (CIP) and its application in wound healing. Reproduced with permission [66]. Copyright 2021, Springer Nature. **b** Schematic diagram of the synthesis of hydrogel films loaded with gentamicin. Reproduced with permission [77]. Copyright 2021 Licensee MDPI, Basel, Switzerland. **c** Schematic diagram of the CMP-Van-Lipo loaded co-gels structure and antibacterial activity. Reproduced with permission [84]. Copyright 2019. Published by Elsevier Ltd

Ciprofloxacin (CIP) is a fluoroquinolone antibiotic with broad antibacterial activity for the treatment of bacterial infections, including respiratory infections, and skin and bone infections [64]. The antibacterial mechanism of CIP is the inhibition of bacterial DNA synthesis and gyrases resulting in bacterial death [65]. Zhu et al. prepared graphene/silk fibroin composite hydrogels and loaded CIP (Fig. 3a) [66]. The antibacterial activities of CIP-loaded graphene/SF hydrogel against *Pseudomonas aeruginosa* (*P. aeruginosa*) and *S. aureus* were detected by the zone of inhibition test. The results suggested that the composite hydrogel could effectively inhibit bacterial growth, accelerate wound healing, and have good release properties. Moreover, this composite hydrogel had good biocompatibility and was nontoxic towards cells, and was suitable for use as wound dressings. Zheng et al. successfully constructed a hybrid hydrogel (Cip-Ti_3_C_2_ TSG) composed of CIP and Ti_3_C_2_ MXene [67]. The results showed that the release of CIP from the hybrid hydrogel could be accelerated under certain conditions, thereby improving the antibacterial efficiency.

Clindamycin is a lincosamide antibiotic widely used for severe skin and soft tissue infections caused by *S. aureus* [68, 69]. Sadeghi et al. used citric acid as a cross-linking agent to cross-link carboxymethyl cellulose and human hair keratin, and loaded clindamycin to prepare a novel antibacterial dressing [70], the results showed that the dressing could effectively inhibit the growth of *S. aureus*, the dressing did not affect the activity of fibroblasts, and the cell survival rate could reach more than 90%. Therefore, it had a good application prospect in the field of skin tissue repair and regeneration. Jiang et al. developed a novel wound dressing based on glycerin hydrogel loaded with clindamycin showing effective antimicrobial properties as well as good biocompatibility [71]. In addition, clindamycin-loaded double cross-linked nanocomposite hydrogel had been reported, which exhibited excellent antibacterial activity against MRSA and *Escherichia coli* (*E. coli*) [72].

Gentamicin belongs to aminoglycoside antibiotics, which is traditional antibiotics with efficient antibacterial activity, and is widely used in the treatment of bacterial infections [73]. If gentamicin acts directly on the skin, its absorption rate is low and it acts only locally on the superficial layer of skin [74]. In addition, gentamicin can also induce nephrotoxicity and ototoxicity, hindering its clinical application [75, 76]. Zhang et al. successfully prepared gentamicin-loaded composite hydrogel (CTMCSG) by reacting CS with epichlorohydrin (Fig. 3b) [77]. The results showed that this novel hydrogel had a good antibacterial effect, and more than 50% of the drug was released within 24 h, indicating its good drug release performance. The above findings suggested that prepared CTMCSG hydrogel had great potential to promote wound healing. Besides, Eltawila et al. reported a new topical antibacterial modality for the topical treatment of mandibular osteomyelitis by using injectable gentamicin-collagen hydrogels [78]. Moreover, gentamicin had been commonly used as antibacterial coatings for orthopedic implants and showed good antibacterial activity [79].

Vancomycin is a glycopeptide antibiotic with a broad antibacterial effect on gram-positive bacteria [80]. Despite great concerns about bacterial resistance and nephrotoxicity, vancomycin remains a clinically important treatment [81]. Liao et al. prepared vancomycin-loaded injectable hydrogels using oxidized hyaluronic acid (HA) and adipic acid dihydrazide [82]. The antibacterial effect of hydrogel against *S. aureus* was evaluated by the disc diffusion method, the results showed that the hydrogels had good antibacterial activity, and the average release rate of vancomycin could reach 86% on the 3rd day, and the as-prepared hydrogels had potential applications in coating the exteriors of orthopedic implants. In addition, vancomycin-loaded dual-function injectable hydrogel could effectively kill *S. aureus* through the pH-responsive sustained release of vancomycin [83]. Besides, collagen hydrogels prepared by Thapa et al. combined with collagen mimetic peptide-conjugated vancomycin liposomes (CMP-Van-Lipo) exhibited strong antibacterial activity (Fig. 3c) and had potential application in treatment of persistent wound infections [84].

In addition to the above antibiotics, the ampicillin-loaded tragacanth nanohydrogels also had inhibitory effects on *E. coli* [85]. Moreover, levofloxacin-loaded HA hydrogel could effectively kill intracellular *S. aureus* and *P. aeruginosa* [86]. From the progress of antibacterial research, antibiotics are still an effective strategy for treating bacterial infections. However, the overuse of antibiotics can lead to the occurrence of drug-resistant bacteria, which seriously endangers human health. Therefore, in addition to the development of new antibiotics, the effective combination of antibiotics and hydrogels can act as a slow-release antibiotic and can be administered directly at the site of infection thereby reducing antibiotic abuse, which is also an effective strategy to address the problem of bacterial resistance.

### Antibiotic-free hydrogels

The adverse consequences caused by the misuse of antibiotics have brought a severe challenge to the medical field [87, 88], antibiotic-free treatment strategies have been actively developed [89]. In contrast to antibiotics, metal ions and metal oxides can enhance and maintain antibacterial activity, and biological extracts, as well as antibacterial polymers, have good antibacterial activity and biocompatibility [90–92]. In general, they can replace the use of antibiotics to reduce the incidence of drug abuse and resistance.

### Antibacterial hydrogels loaded with metal nanoparticles

Hydrogels loaded with metal ions are ideal antibacterial materials due to their broad-spectrum antibacterial effect. The cost and toxicity of metal ions in the use process are still problems that need to be further solved. Hydrogels as carriers for delivery metal ions have good biocompatibility and can provide some strategies for the field of antibacterial therapy.

**Fig. 4:**
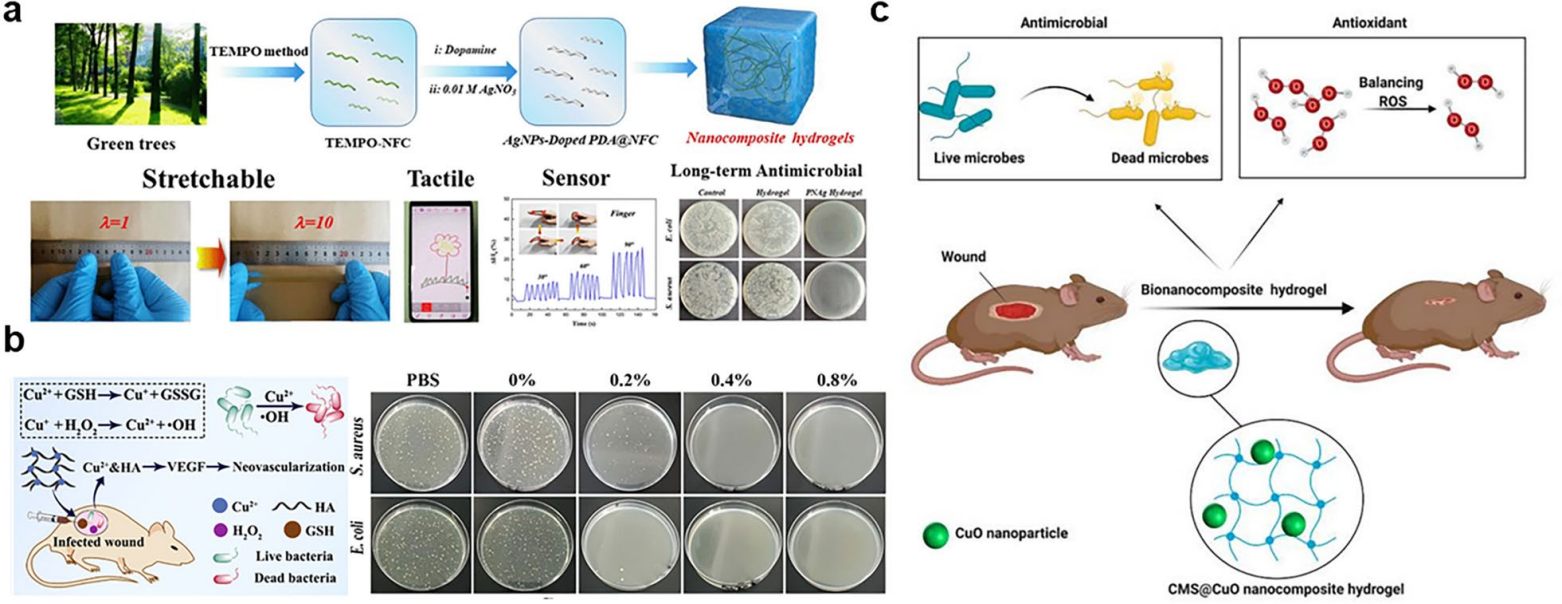
Antibacterial hydrogels incorporated with metal nanoparticles. **a** Preparation process of cellulose composite hydrogel and its characterization and application in sensors. Reproduced with permission [96]. Copyright 2021 Elsevier Ltd. **b** Schematic diagram of antibacterial activity of HA-Cu hydrogel and antibacterial activity of HA-Cu hydrogels with different Cu^2+^ contents. Reproduced with permission [102]. Copyright 2022 American Chemical Society **c** Application of nanocomposite hydrogels containing CuO nanoparticles in wound healing. Reproduced with permission [112]. Copyright 2021 Licensee MDPI, Basel, Switzerland

Metal ions, such as Ag^+^, Cu^2+^, Zn^2+^, Co^2+^, and Au^+^, have broad-spectrum antibacterial effects and are widely used in antibacterial materials [93, 94]. Among them, Ag nanoparticles (AgNPs) are widely used in the field of antibacterial due to their excellent antibacterial properties [95]. Wang et al. introduced covalently cross-linked polyacrylamide (PAM) into AgNPs loaded cellulose networks to prepare a hydrogel sensor with good ductility and antibacterial properties (Fig. 4a) [96]. In vitro antibacterial test results showed that the composite hydrogels had good antibacterial and effective antibacterial adhesion activity. Moreover, Jaiswal et al. prepared the carrageenan-based composite hydrogels incorporated with AgNPs synthesized by using lignin as a reducing agent, which could promote wound healing [97]. The results showed that the composite hydrogels had remarkable antibacterial effects against *S. aureus* and *E. coli*. The composite hydrogels could be used as a wound dressing to promote wound healing in Sprague-Dawley rats. Besides, the composite hydrogel prepared from marine-derived polysaccharides, such as sodium alginate and CS, and AgNPs loaded in composite hydrogels showed good antibacterial activity and had good application prospects in the field of antibacterial dressings [98].

Copper-based nanoparticles have also received much attention from researchers. Compared with silver-based nanoparticles, copper nanoparticles have the advantages of lower cost, easy release in vivo, and remarkable antibacterial properties [99]. Copper nanoparticles are an excellent alternative to silver in cell imaging and photothermal therapy [100, 101]. Qian et al. developed the HA-Cu hydrogel by coordinating hydrazide HA and Cu^2+^ as illustrated in Fig. 4b [102], which had good biocompatibility and significant antibacterial activity against *E. coli* and *S. aureus*. The wound-healing ability of the HA-Cu hydrogel was tested in a rat skin full-thickness model, and the results showed that the hydrogel could significantly accelerate wound healing. Besides, the copper-based antibacterial hydrogel had potential applications not only in wound healing but also in tissue engineering. Furthermore, Qi et al. prepared composite hydrogel for bone defect repair by cross-linking alginate with Cu^2+^ and cannabidiol [103]. The results showed that the hydrogel had an obvious inhibitory effect on *S. aureus* and *E. coli*, and effectively enhanced bone cell differentiation and inhibited inflammatory responses. In addition, Malagurski et al. produced Cu-alginate hydrogel by electrostatic extrusion, which showed immediate bactericidal effects on *S. aureus* and *E. coli* [104].

In addition to the above-mentioned metal nanoparticles, gold nanoparticles (AuNPs) and Zn nanoparticles also exhibited good antibacterial activity. Li et al. designed molybdenum disulfide nanosheets burdened with bovine serum albumin-modified AuNPs, which were then anchored onto an injectable hydrogel [105]. The as-prepared hydrogel could promote wound healing by consuming glucose, scavenging bacteria, and reactive oxygen species. In addition, Tao et al. prepared composite hydrogel through the polymerization of the gelatin methacrylate and dopamine methacrylate, followed by metal coordination between zinc ions and dopamine methacrylate [106]. The reactive oxygen species generated by the composite hydrogel had obvious antibacterial effects on *S. aureus* and *E. coli*. At the same time, it could also stimulate wound healing and promote collagen deposition, which provided a new strategy for the development of wound dressings.

### Antibacterial hydrogels loaded with metal oxide nanoparticles

In addition to the good antibacterial properties of metal nanoparticles combined with hydrogels, the hydrogels loaded with metal oxides also showed outstanding antibacterial properties. And similar to metal ions, metal oxides also have low resistance and broad-spectrum antibacterial effects but metal oxide nanoparticles are more stable [107]. The disadvantages of metal oxides the loaded hydrogels are that metal oxides are not easily released, and have some long-term toxicity and environmental cumulative toxicity. However, the combination of hydrogels with metal oxides can solve the problem of toxicity accumulation and reduce the occurrence of antibiotic resistance, as well as achieve a better antibacterial effect to some extent.

Among various metal oxides, zinc oxide nanocomposite (ZnO NPs) has been reported has been reported to have antibacterial activity with low cytotoxicity [108]. Kummara et al. prepared HA-ZnO NPs composite hydrogel with a one-pot synthesis method [109]. The results of the plate diffusion method showed that the composite hydrogel exhibited excellent antibacterial activity, and the antibacterial activity against *E. coli* was better than that of *S. aureus*. The composite hydrogel also exhibited good biocompatibility and other properties, which indicated its potential application in wound dressings. Besides, Tantiwatcharothai et al. used basil seed mucilage and ZnO NPs to prepare bacterial wound dressings by freeze-drying [110]. The results showed that the hydrogel exhibited significant antibacterial properties and water retention capacity with low cytotoxicity.

Another metal oxide nanoparticle copper oxide nanoparticles (CuO NPs), is easy to mix with polymers to prepare hydrogels with relatively stable physical and chemical properties [111]. Thus, copper-based nanocomposites have attracted much attention. Abdollahi et al. combined sodium carboxymethylated starch and CuO NPs to prepare nanocomposite hydrogel using citric acid as a cross-linking agent as illustrated in Fig. 4c [112]. The experimental results showed that the composite hydrogel had an inhibitory effect on eight kinds of bacteria that were pathogenic to humans. The composite hydrogel that contained 2 wt.% CuO NPs was low cytotoxicity against human fibroblasts. At the same time, the results of in vitro experiments showed that the composite hydrogel could accelerate wound healing. Besides, Wahid et al. prepared carboxymethyl CS/CuO composite hydrogel based on CuO NPs [113]. The results of the colony forming unit method showed that the composite hydrogel exhibited excellent antibacterial activity against *S. aureus* and* E. coli*.

In addition to the above-mentioned metal oxide nanoparticles, poly (HEA-AAm)/WO_3_ hydrogel incorporating WO_3_ nanoparticles had been demonstrated to possess antibacterial activity against bacteria causing bacterial keratitis, such as *E. coli*, *P. aeruginosa*, and* Candida albicans* [114]. Zhang et al. prepared a novel TiO_2_ NPs-β-cyclodextrin-cellulose composite hydrogel that showed high antibacterial activity against *E. coli*. and *S. aureus* under light condition [115]. Meanwhile, the drug sustained-release experiment showed that hydrogel could completely release curcumin after 120 h. In addition, Hanan Albalwi et al. incorporated magnesium oxide nanoparticles (MgO NPs) into acrylic acid/polyvinyl alcohol (PAA/PVA) hydrogel by using γ-radiation technique, which exhibited effective antibacterial effect as the concentration of MgO NPs increased [116].

### Antibacterial hydrogels loaded with antibacterial polymers

In recent years, antibacterial polymers have attracted much attention in antibacterial materials due to their high availability, good biocompatibility, and easy degradation [117, 118]. Therefore, the use of antibacterial polymers can enhance the antibacterial efficiency of existing antibacterial agents, and help to delay the occurrence of bacterial resistance [119]. Covalent attachment of antibacterial polymers to hydrogels enhanced the steadiness and dispersibility of polymers and improved the properties of hydrogels. Therefore, hydrogels loaded with antibacterial polymers have great potential applications in the antibacterial field. Here, we would introduce several classes of typical antibacterial polymers.

CS, a natural linear polysaccharide derived from the exoskeleton of crustaceans, exhibits excellent biological activities including antibacterial activity and wound healing promotion [120, 121]. Meanwhile, CS has been widely used because of its excellent antibacterial properties and good biocompatibility (Table 1). For example, Lucretia et al. reported hydrogel scaffolds cross-linked by CS and fibrin [122]. The result showed that the scaffold had antibacterial activity against *Enterococcus faecalis*, and could promote a bacteria-free environment in the endodontic space. Quaternary ammonium treatment or modified by amino graft hydrophobic alkyl could enhance the antibacterial activity of CS [123]. In addition, He et al. successfully synthesized quaternary ammonium CS-*g*-poly(acrylic acid-co-acrylamide) superabsorbent hydrogel [124]. With the introduction of quaternary ammonium chitosan (QCS), the antibacterial activity of the hydrogel against *E. coli* and *S. aureus* was enhanced. Hydrogel formed by cross-linking of quaternary ammonium chitosan/carboxymethyl starch/alginate can effectively inhibit bacterial growth and achieve rapid hemostasis, which has great prospects for application as a hemostatic antibacterial material [125]. Moreover, Liu et al. developed a nano-antibacterial hydrogel based on QCS, which had a good antibacterial effect against methicillin-resistant *S. aureus*, *E. coli*, and vancomycin-resistant *Staphylococcus* and accelerated wound healing [126]. Furthermore, the composite hydrogel prepared from carboxymethyl cellulose and QCS demonstrated a significant inhibitory effect on bacterial growth [127].

**Table 1:** Representative examples of chitosan-based hydrogels

Chitosan-based hydrogels	Bacterial	Applications	References
Modified CS hydrogel	*Staphylococcus*	Antibacterial therapy	[128–130]
Atechol and methacrylate modified CS-gelatin hydrogel	*P. aeruginosa* *S. aureus*	Wound treatment	[131, 132]
CS- polyethylene glycol (PEG) hydrogel	*E. coli* *S. aureus*	Antibacterial therapy	[133, 134]
Cellulose-CS hydrogel	*E. coli* *S. aureus*	Antibacterial therapy	[135–137]
CS-sodium alginate hydrogel	*E. coli* *S. aureus*	Lysozyme delivery and antibacterial therapy	[138, 139]
CS-L-arginine hydrogel	*E. coli* *S. aureus*	Wound treatment	[140]
CS-dopamine hydrogel	*S. aureus*	Wound dressing	[141]
CS- PVA hydrogel	*E. coli* *S. aureus*	Antibacterial therapy	[142, 143]

Polylysine (PLL) can inhibit bacterial growth by inducing binding to negatively charged bacterial surfaces through positive surface charge [144]. Zou et al. prepared a composite hydrogel based on PLL, in which PLL can be released rapidly to achieve high bactericidal properties [145]. In addition, PLL takes advantages of good biocompatibility, excellent tissue adhesion and anti-infection properties, which widely used in biomedical applications such as wound dressings and biological adhesives [146, 147]. Sun et al. prepared injectable hydrogels based on glycidyl methacrylate and PLL, which had broad-spectrum antimicrobial properties as well as good biocompatibility [148]. The results showed that the hydrogel had an inhibitory effect on *S. aureus* and *E. coli* as well as promoted wound healing in in vivo infection model, which had potential applications in the field of anti-infection and wound healing. And, Ran et al. developed with calcium ions as coagulant a multifunctional composite hydrogel composed of poly (glutamic acid) and PLL, which has good antibacterial properties, adhesion and hemostatic effect [149]. The novel bio-adhesive hydrogel was prepared by mixing HA and PLL [150]. The results showed that the hydrogel was effective in killing *E. coli* and *S. aureus* and reduced the wound healing time due to the large amount of positively charged on the surface. In another study, a hydrogel was prepared from HA and PLL, which had good efficacy in preventing wound infection and promoting wound healing [151].

Polyethyleneimine (PEI) can bind to negatively charged bacteria and eventually lead to bacterial lysis and death [152]. Meng et al. reported a viscous hydrogel antibacterial coating composed of polydextran aldehyde and PEI, which effectively inhibited the growth of *E. coli*, *S. aureus*, and *P. aeruginosa* [153]. And Ren et al. also developed a composite bioinspired coating based on PEI and TA, which exhibited excellent antifogging and antibacterial properties [154]. In addition, the delivery of exosomes using bioactive dressings PEI was a potential strategy for the repair of diabetic wounds. Wang et al. prepared an injectable hydrogel scaffold that was composed of Pluronic F127 grafted PEI and aldehyde pullulan and loaded with exosomes [155], which had excellent antibacterial activity and rapid hemostasis and could release exosomes, promote angiogenesis and wound healing. In addition, by modifying polymers with PEI, antibacterial properties could be introduced into polymers that lack antibacterial properties. The antibacterial hydrogel was prepared from bacterial cellulose and PEI by using epichlorohydrin as a cross-linking agent[156]. Studies have shown that hydrogel had excellent antibacterial effects against *S. aureus* and *E. coli*.

### Antibacterial hydrogels loaded with peptides

**Fig. 5:**
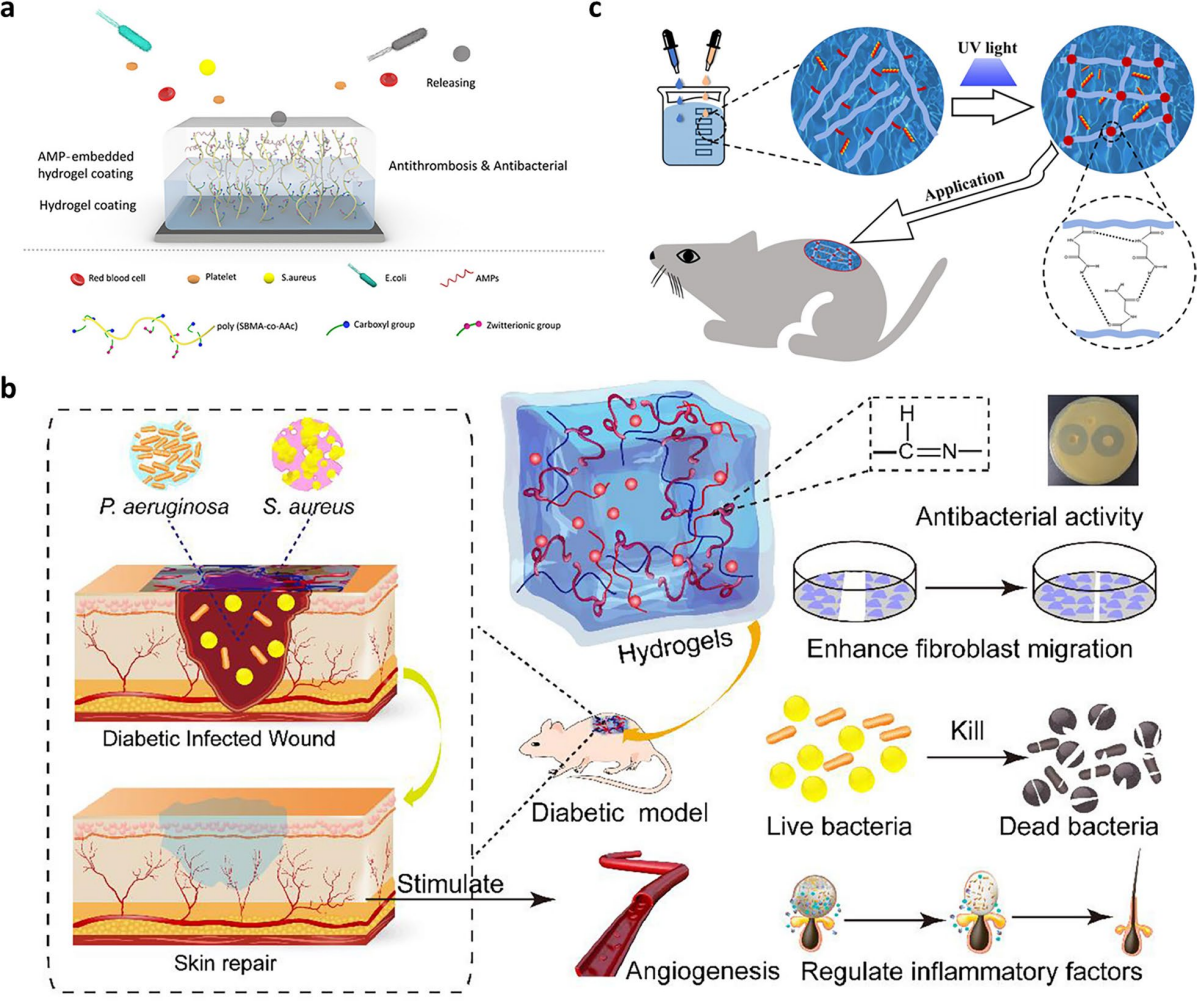
Antibacterial hydrogel incorporating antimicrobial peptides. **a** Hydrogel coating embedded with antibacterial peptides. Reproduced with permission [159]. Copyright 2021 American Chemical Society. **b** Schematic diagram of the synthesis of a hydrogel dressing that can be used for diabetic wound healing. Reproduced with permission [161]. Copyright 2021 Elsevier Ltd. **c** Schematic diagram of the preparation of self-fusing supramolecular hydrogels and their applications. Reproduced with permission [162]. Copyright 2022 IOP Publishing

Antimicrobial peptides (AMPs) is a kind of cationic short peptide substance, which shows strong antibacterial activity against gram-negative and gram-positive bacteria, viruses, and fungi [157]. The antibacterial mechanism of AMPs is to combine with the bacterial cell membrane and destroy the cell membrane to kill bacteria [158]. It is worth mentioning that the antibacterial mechanism of AMPs is not easy to cause the emergence of drug-resistant bacteria. Therefore, AMPs provide new ideas for solving the problem of traditional antibiotic resistance. Liu et al. developed an AMP-embedded hydrogel coating using sulfobetaine methacrylate and acrylic acid as hydrogel monomers (Fig. 5a) [159]. The AMPs with two different amino acid residues were embedded in the hydrogel coating by chemical grafting. The results showed that the coating had excellent antibacterial against gram-negative bacteria and gram-positive bacteria and antithrombotic properties. In addition, AMPs not only exhibited excellent antibacterial activity but also had potential applications in the field of wound healing [160]. Wei et al. prepared a composite hydrogel formed by Schiff base linkage that was composed of oxidized dextran, platelet-rich plasma, HA, and cecropin of a short peptide of 23 amino acids as illustrated in Fig. 5b [161]. The results showed that the composite hydrogel exhibited an obvious inhibitory effect on three pathogenic bacteria and could promote wound healing in diabetic mice. Wang et al. developed polymer hydrogel composed of polymerized N-acryloyl glycinamide monomer and introduced it with the antibacterial peptide polymyxin E, the hydrogel (PN-AP hydrogel) showed antibacterial effects against *E. coli* and *S. aureus* and showed good results in promoting wound healing(Fig. 5c) [162].

### Antibacterial hydrogels loaded with plant extracts

The antibacterial effect of plant extracts has been an important research subject [163]. Facing with the threat of multidrug-resistant bacteria, the antibacterial effect of plant extracts provided new ideas for treatment methods and the development of novel antibacterial agents, which brings hope to reduce the occurrence of drug-resistant bacteria [164, 165]. At the same time, plant extracts had the advantages of wide source, easy extraction, and low toxicity as well as good antibacterial properties, which provided a new attempt for the development of new antibacterial agents and application in antibacterial therapy [165]. In addition, the combination of plant extracts and hydrogel could improve their solubility and easy release, which was of great significance in antibacterial applications. Many essential oils (EOs) including thyme oil, peppermint, tea tree, cinnamon, lemongrass, and eucalyptus had been proven to exhibit antibacterial properties [166–169]. Hybrid EOs loaded with carbomer hydrogel had been reported to have good antibacterial activity against *S. aureus*, *E. coli*, and *P. aeruginosa*[170]. Feng et al. prepared the composite hydrogel containing TA that had good antibacterial activity against *S. aureus* and had potential application in wound dressings[143].

### The stimuli-responsive smart antibacterial hydrogels

The stimuli-responsive hydrogels are capable of dramatic volume change in response to changes in the environment to which they are exposed, such as pH, temperature, and light [171, 172]. Environmental conditions determine sol-gel phase transition behavior. Compared with normal tissues, the infected microenvironment is characterized by lower pH, higher local temperature, and higher content of secreted enzymes [173, 174]. Moreover, the acidic infected microenvironment can act as a switch to deliver stimuli-responsive drugs [175]. Due to the difference between infected and healthy sites, the smart responsive hydrogels can target the delivery of drugs or directly activate their antibacterial activities, which will improve the utilization of drugs and reduce the occurrence of drug-resistant bacteria [176]. At the same time, the smart responsive hydrogels also provide new therapeutic strategies for infection treatment. For example, Liu et al. developed a novel hydrogel that consisted of reversible catechol-boronate linkage and pH-responsive between chlorinated catechol and phenylboronic acid [177]. The results showed that the hydrogel exhibited good antibacterial activity against gram-positive bacteria and gram-negative bacteria including MRSA under acidic conditions. On the contrary, under alkaline conditions, the hydrogel had no antibacterial effect. In addition, the pH-sensitive hydrogels had potential applications in wound dressings for wound healing and drug delivery [178, 179]. In addition to pH-sensitive hydrogel, many hydrogels consisting of poly(N-isopropyl acrylamide) (PNIPAM) copolymers were thermally responsive due to the critical solution temperature of PNIPAM in the aqueous solution of about 33 °C, which exhibited excellent thermally responsive self-shrinkage properties at body temperature [180]. PNIPAM-based thermal response hydrogel was reported that had good antibacterial activity, and biocompatibility, and could assist wound closure and promote wound healing [181]. In another study, thermos-responsive hydrogels were synthesized using Pluronic F127 and cellulose, which showed good antimicrobial properties [182]. In addition to the above two intelligent response types, enzyme response and oxidation restore response also reflected good bacteriostatic effects and the performance of promoting wound recovery [183, 184].

### Light-mediated antibacterial hydrogels

#### Photothermal antibacterial hydrogels

**Fig. 6:**
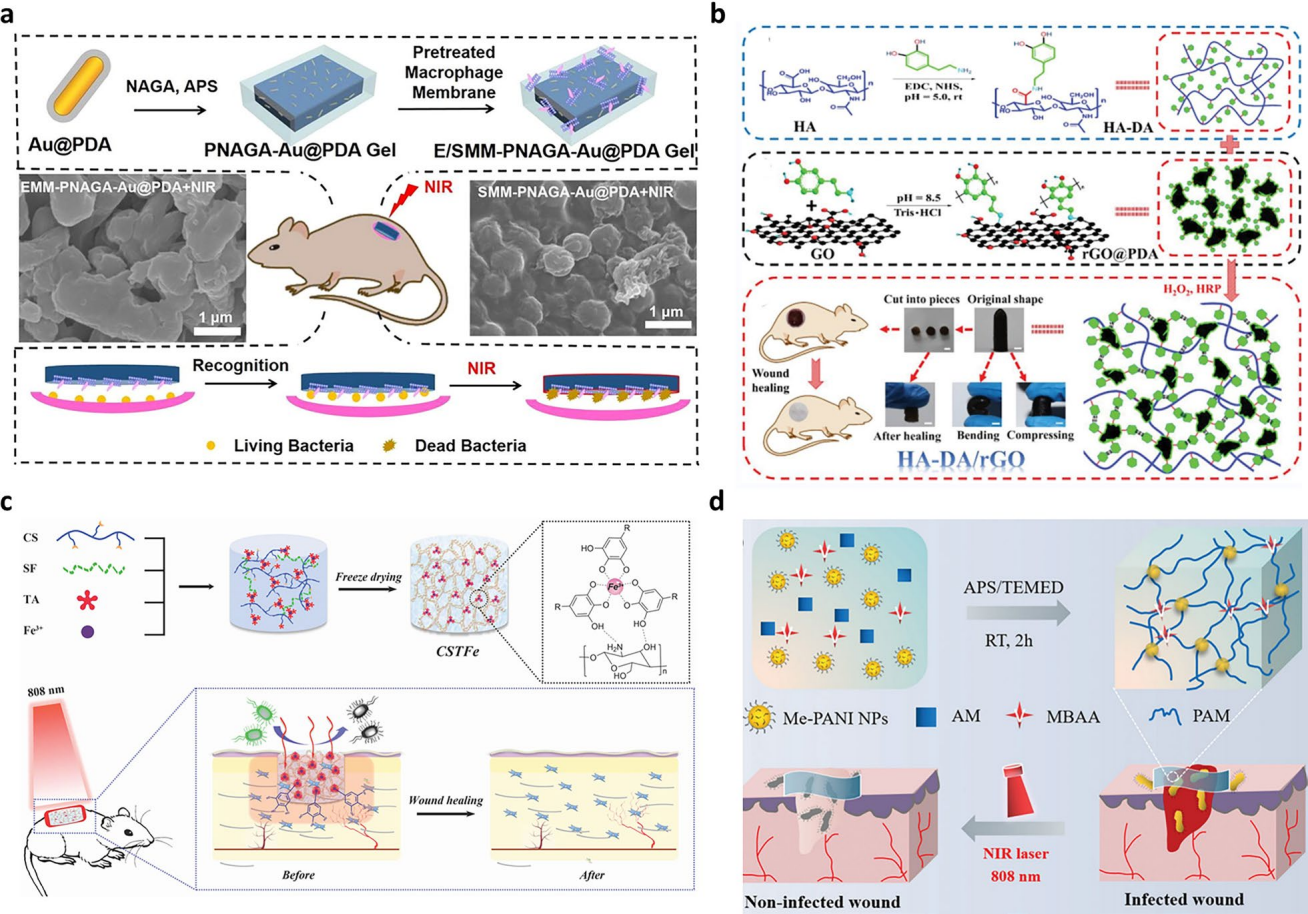
Photothermal antibacterial hydrogels. **a** Preparation process of E/SMM-PNAGA-Au@PDA NRs hydrogel and its application in wound healing. Reproduced with permission [191]. Copyright 2020 Elsevier B.V. **b** Synthesis strategy of HA-DA/rGO hydrogel, shape, and healing under mechanical force and schematic diagram of its application in wound healing. Reproduced with permission [193]. Copyright 2019 John Wiley & Sons, Inc. **c** Schematic diagram of the preparation process of hydrogel and its application in promoting wound healing. Reproduced with permission [195]. Copyright 2019 John Wiley & Sons, Inc. **d** Structure of Me-PANI NPs@PAM hydrogels and their application in wound infection. Reproduced with permission [197]. Copyright 2021 Wiley–VCH GmbH

Photothermal therapy (PTT) is a highly effective multidrug-resistant treatment strategy with advantages such as low systemic toxicity, minimal invasiveness, low drug resistance, spatiotemporally control, and negligible side effects [185, 186]. As at the infectious site, the thermal effect caused by PTT can accelerate the release of the drug [187]. PTT shows high efficiency and low-drug resistance, which provides a new way for the treatment of drug-resistant bacterial infections. Although the antibacterial activity of photothermal nanomaterials is considerable, further evaluation of their long-term biocompatibility is needed. The exploration of PTT is still at the early stages, but it has potential applications in the antibacterial field. Photothermal agents are essential elements in PTT, which can convert light into heat, causing protein-denaturing bacteria to lyse and die as well as inhibiting the formation of the biofilm [188, 189]. Precious metal materials are widely applied as photothermal agents. Among them, AuNPs are used in photothermal sterilization due to their good biocompatibility and high photothermal conversion rate [190]. Li et al. developed a composite hydrogel by mixing monomer N-acryloyl glycinamide (NAGA) with polydopamine-coated gold nanorods (Au@PDA NRs). And then, *E. coli* or *S. aureus*-pretreated macrophage membrane coat the PNAGA-Au@PDA hydrogel termed as E/SMM-PNAGA-Au@PDA (Fig. 6a) [191]. The results showed that the hydrogel could not only specifically recognize bacteria, but also had a rapid antibacterial effect on *S. aureus* or *E. coli* under near-infrared radiation (NIR) and killed 98% of bacteria for 5 min.

In addition, carbon-based nanomaterials including graphene derivatives and carbon nanotubes have also attracted much attention in photothermal therapy. Han et al. prepared graphene oxide-containing composite hydrogel that had significant antibacterial effects against *S. aureus* and *E. coli* under NIR [192]. Also, Liang et al. prepared antibacterial hydrogel based on HA-grafted dopamine and graphene oxide for wound dressings (Fig. 6b) [193]. The results showed that the hydrogel exhibited good photothermal properties and antibacterial effects under NIR. At the same time, the hydrogel significantly promoted angiogenesis and facilitates collagen deposition, thereby accelerating wound healing in rats. Besides, metal coordination complexes composed of catechol and iron ions have been reported to have good photothermal efficiency due to the absorption of NIR light from 650 to 1350 nm [194]. Yu et al. developed cryogel with TA/iron ions as photothermal agents and CS/silk fibroin as the scaffolds, which exhibited good antibacterial properties and hemostatic ability and could promote wound healing (Fig. 6c) [195]. In addition to precious metals and inorganic non-metallic materials, some organic materials are also used in photothermal hydrogel for antibacterial. Polyaniline (PANI) is a conjugated polymer widely applied in PTT due to its high photothermal conversion rate [196]. Pang et al. developed the novel nanoparticles cross-linked hydrogel (Me-PANI NPs@PAM) by grafting PANI onto methacrylated ethylene glycol CS and then cross-linking with PAM as illustrated in Fig. 6d [197]. The in vitro research showed that the hydrogel could effectively inhibit the growth of *S. aureus* upon NIR and accelerate wound healing.

### Photodynamic antibacterial hydrogels

Photodynamic therapy (PDT) is also an effective antibacterial method, which is a non-invasive antibacterial and anti-biofilm treatment strategy [198]. PDT relies on photosensitizers (PSs) to generate reactive oxygen species (ROS) under the light of appropriate wavelength, which ruptures bacterial cell membranes, inactivates DNA and proteins, and achieves the purpose of sterilization [199, 200]. However, hydrophobicity and poor water solubility of most PSs require further in-depth research and resolution. With the increasing occurrence of drug-resistant bacteria, PDT, as a broad-spectrum antibacterial therapy, has gradually become one of the therapeutic strategies for the treatment of bacterial infections. He et al. prepared a multifunctional hydrogel antibacterial coating based on methacrylate gelatin encapsulated mesoporous polydopamine nanoparticles, which loaded the photosensitizer Chlorin E6 through the π-π stacking reactions [201]. When the laser irradiation is 660 nm, the coating had been demonstrated to have the ability to the same time antibacterial and promote fibroblast activation. And under 1 W cm^−2^ irradiation power, the coating could rapidly eliminate bacteria. Besides, Zhang et al. developed the photodynamic hydrogel based on a small peptide and a fullerene, which demonstrated the ability to target and sustain antibacterial therapy [202]. The results in vitro and in vivo antibacterial experiments proved that the hydrogel could effectively inhibit *S. aureus* growth and promote wound healing. It is worth mentioning that compared with gram-positive bacteria, gram-negative bacteria have a thicker outer membrane, which makes some PS cannot effectively kill bacteria [203]. In addition, Bayat et al. developed a photodynamic antibacterial hydrogel using zinc phthalocyanine-colistin conjugate as a PS [204]. The binding of zinc phthalocyanine to colistin enhances the permeability of the outer membrane of gram-negative bacteria. The results showed that the photodynamic antibacterial hydrogel had an antibacterial effect on *P. aeruginosa*.

**Fig. 7:**
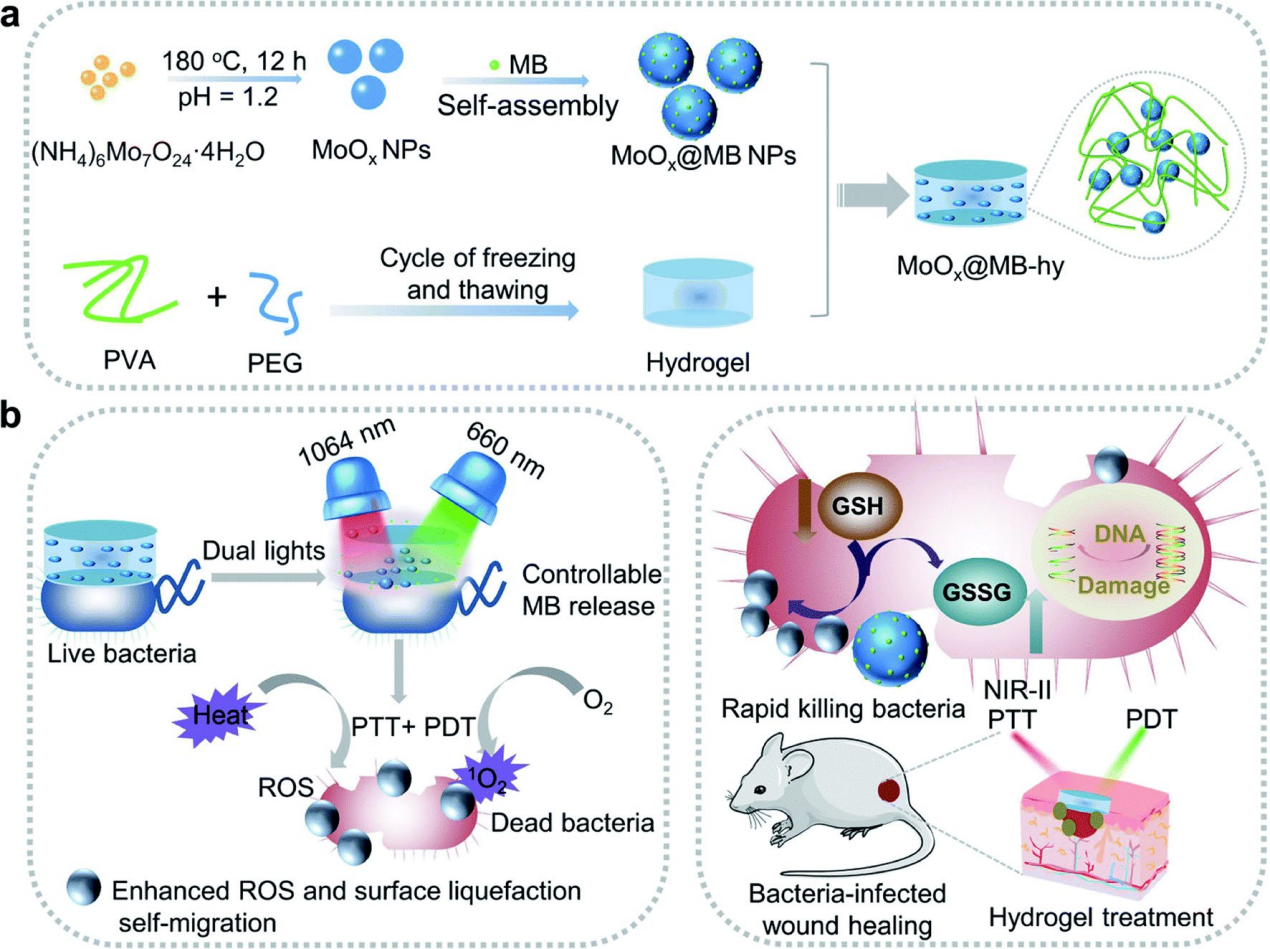
Photodynamic and photothermal synergistic antibacterial hydrogels. **a** Synthesis process of MoOx@MB-hy hydrogel. **b** Schematic diagram of PTT-PDT synergistic antibacterial effect. Reproduced with permission [207]. Copyright 2022 The Royal Society of Chemistry

With the increasing threat of drug-resistant bacteria, the advantages of PDT and PTT, such as controllability, open new strategies against drug-resistant bacteria. The combination of PTT and PDT has a more significant bacteriostatic effect compared with single antibacterial therapy. The thermal energy generated under the action of PTT can damage the cell membrane, while the ROS generated under the action of PDT can leak proteins to further achieve a bactericidal effect [205, 206]. Therefore, the combination of PDT and PTT can not only enhance the antibacterial efficiency but also reduce the occurrence of side effects, which has potential applications in the field of drug-resistant bacteria treatment. Wang et al. prepared a nanocomposite hydrogel (MoO*x*@MB-hy) consisting of PVA and PEG loaded with defective-structured molybdenum oxide nanoparticles (MoOx NPs) and PS methylene blue (MB) (Fig. 7a) [207]. Under the dual irradiation of NIR and 660 nm laser, the composite hydrogel could break the glutathione antioxidant balance and accumulate ROS. The results showed that dual-light irradiation could effectively eliminate *E. coli* and *Bacillus subtilis* (*B. subtilis*) within 15 min (Fig. 7b). Meanwhile, in vitro experiments showed that hydrogel could accelerate the healing of *E. coli*-infected wounds. In another study, they fabricated 3D CuS@MoS_2_ microspheres using MoS_2_ and CuS and then incorporated them into PVA hydrogel to obtain bifunctional hydrogel [208]. The hydrogel could rapidly generate ROS under the co-irradiation of 660 nm visible light and 808 nm near-infrared light, and exhibited a remarkable antibacterial efficiency of over 99% against both *S. aureus* and *E. coli*. In addition, CuS@MoS_2_ could promote the secretion of the hypoxia-inducible factor-1 (HIF-1) and vascular endothelial growth factor (VEGF) to promote the proliferation of endothelial cells. Meanwhile, some new injectable hydrogels had also shown good antibacterial effects under the dual action of PTT and PDT, and could be used as candidates for wound dressings [209, 210]. Under the action of both PTT and PDT methods, the antibacterial efficiency can be improved. The thermal energy generated by PTT can reduce bacterial activity, while PDT can also make bacteria more sensitive. Therefore, the combination of the two methods can effectively overcome the shortcomings of the other method and has great potential in the application of antibacterial therapy.

### Antibacterial hydrogels with synergistic effects

Hydrogels with synergetic effects can enhance the antibacterial effect under the action of two or more antibacterial modes [211]. Incorporating two and more types of antibacterial agents (usually metal nanoparticles and antibiotics) into hydrogel has been reported to produce synergistic antibacterial effects. Synergistic effective hydrogel incorporating metal nanoparticles shows efficient antibacterial ability and wide spectrum antibacterial effect. In addition, combined use with antibiotics is an excellent way to reduce the occurrence of drug-resistant bacteria and reduce the abuse of antibiotics. Compared with a single antibacterial agent, the incorporation of two or more antibacterial agents into the hydrogel can produce a synergistic effect, which can expand the antibacterial spectrum and enhance the antibacterial effect. Due to the different antibacterial mechanisms of various antibacterial agents, the synergistic hydrogel is not easy to cause the occurrence of drug-resistant bacteria, which has certain application prospects in the treatment of infection. Among them, AgNPs are most used in combination with antibiotics. AgNPs have good biocompatibility and antibacterial effect, which are more suitable for use in biomedicine [212]. Yu et al. developed an hydrogel antibacterial coating doped with HA/AgNPs/gentamicin, which exhibited good antibacterial activity and good biocompatibility [213]. Not only AgNPs and antibiotics showed excellent antibacterial ability, but AgNPs and other antibacterial agents together also had amazing antibacterial effects. Shome et al. developed an antibacterial composite hydrogel containing AgNPs based on amino acid in situ self-assembly [214]. The composite hydrogel exhibited lethal killing effects on both gram-negative bacteria and gram-positive bacteria and had strong biocompatibility, which had a variety of potential applications in the field of tissue engineering. In addition, the composite hydrogel prepared by combining AgNPs with biological extracts have promising applications in antibacterial therapy. Liu et al. prepared composite hydrogels introducing AgNPs and aloe vera, which further enhanced the antibacterial effect and showed a synergistic effect [215]. The composite hydrogel not only exhibited good antibacterial properties and biocompatibility but also promoted cell proliferation and wound healing, which had potential application as wound dressings. Anjum et al. reported that the composite hydrogel containing AgNPs, aloe vera, and curcumin could exhibit a good inhibitory effect on *S. aureus* and *E. coli* while promoting rapid wound healing [216]. Furthermore, the incorporation of AgNPs into CS-based hydrogel was also an effective method to enhance the antibacterial effect. Masood et al. reported that CS-PEG-AgNPs based on hydrogel could slowly release silver nanoparticles, which had certain applications in the treatment of chronic diabetic wounds [217]. The experimental results showed that the composite hydrogel exhibited inhibitory effect on *S. aureus*, *B. subtilis*, *P. aeruginosa*, and *E. coli*. Some researchers prepared composite antibacterial hydrogel based on CS-g-PAM and loaded with AgNPs synthesized using Curcuma longa [218]. The results could prove that the composite hydrogel had high antibacterial activity against *S. aureus* and *E. coli*, and the antibacterial rate could reach 99.99%.

Among the synergistic hydrogels, in addition to the metal nanoparticles showing good antibacterial activity, the antibiotic-loaded hydrogels combined with other antibacterial materials also showed highly efficient antibacterial effect and good biocompatibility. Yan et al. combined CS and gentamicin to prepare a scald dressing with antibacterial, anti-inflammatory and promoting wound healing [219]. In another study, Gezgin et al. incorporated gentamicin and propolis extracts in a thermosensitive hydrogel, which exhibited good antibacterial properties against MRSA [220]. In addition to gentamicin, Yang et al. prepared an injectable hydrogel composed of PEI, tobramycin and chondroitin sulfate by Schiff base reaction, which showed effective antibacterial effect and had potential application as wound dressings [221]. The hydrogel doped with CIP microspheres and ginsenosides also exhibited good antibacterial activity against *S. aureus* [222]. The addition of vitamin E and levofloxacin to hydrogel contact lenses not only treated keratitis caused by bacteria but also prolonged the release of antibiotics, which had potential applications for treating bacterial infections [223].

## Conclusion and prospect

This review summarized the latest application progress of antibacterial hydrogels in the biomedical field. The fabrication process of the hydrogels and the hydrogels loaded with various antibacterial agents were described in detail. Hydrogels incorporated with antibacterial drugs and antibacterial polymers exhibited good antibacterial activity. Furthermore, embedding inorganic antibacterial agents, including metal nanoparticles and graphene derivatives materials, into hydrogels also exhibited promising antibacterial activity [224]. The matrices of the hybrid hydrogels could use various organics, including natural polymers and man-made polymer materials, which demonstrated the biocompatibility of antibacterial agents and controlled as well as the sustained release of antibacterial agents. It is worth mentioning that some antibacterial agents could enhance the mechanical properties of the hydrogels, including strength and toughness while imparting antibacterial properties to the hydrogel [225, 226]. At present, the antibacterial hydrogels are widely used in wound dressings to treat wound infections, contact lenses, urinary tract coatings, catheter-associated infections, gastrointestinal infections, treatment of osteomyelitis, and so on [90, 227–231]. Due to their excellent biocompatibility, good physical and chemical properties, the hydrogels can provide new therapeutic strategies as carriers of antibiotics or replace antibiotics relying on their inherent antibacterial effects. At the same time, the antibacterial hydrogels can not only be used for local treatment but also can be used as a drug delivery system to deliver drugs that achieve sustainable drug release and long-term antibacterial purposes. The hydrogels can carry a variety of antibacterial agents, and the antibacterial component can play the role in synergistic treatment to enhance the antibacterial effect. Therefore, as a novel type of antibacterial biomaterial, the hydrogels can have broader antibacterial effects and better antibacterial properties, which can further promote the development of anti-infective therapy.

Hydrogels loaded with antibiotics and metal nanoparticles show good antibacterial properties in treating infections and fighting biofilms. However, metal nanoparticles have problems of poor biocompatibility and high cytotoxicity, and antibiotics are susceptible to resistance, which greatly limit their application in the biomedical field. It is worth noting that the occurrence and evolution of drug-resistant bacteria are mostly caused by the abuse of antibiotics [232]. The introduction of new antibacterial strategies has significantly reduced the incidence of drug-resistant bacteria. PTT has been widely applied in antibacterial applications. In addition to exhibiting good bactericidal effects in PTT, ultrasound, and magnetic fields can also be utilized to treat bacterial infections [233]. This is expected to provide a useful case for the research and application of antibacterial hydrogels. In addition, cross-linking agents are also worthy of our attention. The chemical cross-linking agents usually used in the preparation of hydrogel are cytotoxic and harmful to the environment. Therefore, the development and use of green and non-toxic cross-linking agents and preparation methods still require further research. With the continuous development of research, we believe that there will be relevant strategies to solve these problems to prepare greener, safer, and more efficient antibacterial hydrogels.

The key to future research directions is to continuously improve the antibacterial performance of hydrogels against multidrug-resistant bacteria. As research continues, hydrogels have a wide range of antibacterial activity, which makes them potentially useful in the treatment of bacterial infections. We believe that the hydrogels as antibacterial biomaterials will provide broad application prospects for anti‐infection treatment through their unique combinations and continuous development.

## Data Availability

Not applicable.
